# Developing a machine learning model to predict patient need for computed tomography imaging in the emergency department

**DOI:** 10.1371/journal.pone.0278229

**Published:** 2022-12-15

**Authors:** Amirmohammad Shahbandegan, Vijay Mago, Amer Alaref, Christian B. van der Pol, David W. Savage

**Affiliations:** 1 Department of Computer Science, Lakehead University, Thunder Bay, Ontario, Canada; 2 NOSM University, Thunder Bay, Ontario, Canada; 3 Department of Diagnostic Imaging, Juravinski Hospital and Cancer Centre, Hamilton, Ontario, Canada; University of Wisconsin-Eau Claire, UNITED STATES

## Abstract

Overcrowding is a well-known problem in hospitals and emergency departments (ED) that can negatively impact patients and staff. This study aims to present a machine learning model to detect a patient’s need for a Computed Tomography (CT) exam in the emergency department at the earliest possible time. The data for this work was collected from ED at Thunder Bay Regional Health Sciences Centre over one year (05/2016-05/2017) and contained administrative triage information. The target outcome was whether or not a patient required a CT exam. Multiple combinations of text embedding methods, machine learning algorithms, and data resampling methods were experimented with to find the optimal model for this task. The final model was trained with 81, 118 visits and tested on a hold-out test set with a size of 9, 013 visits. The best model achieved a ROC AUC score of 0.86 and had a sensitivity of 87.3% and specificity of 70.9%. The most important factors that led to a CT scan order were found to be chief complaint, treatment area, and triage acuity. The proposed model was able to successfully identify patients needing a CT using administrative triage data that is available at the initial stage of a patient’s arrival. By determining that a CT scan is needed early in the patient’s visit, the ED can allocate resources to ensure these investigations are completed quickly and patient flow is maintained to reduce overcrowding.

## Introduction

Overcrowding in the emergency department (ED) is a well-documented worldwide phenomenon [1]. The ED is an essential point of entry to the healthcare system for patients who require urgent care. Delays in the ED can lead to potentially harmful effects including increased mortality and morbidity [2]. Overcrowding occurs when limited resources cause delays in the ED while patients are waiting to receive care [3]. The causes of overcrowding are numerous and interconnected including increased patient volume, lack of beds for admitted patients, delays in services provided by diagnostic services (*e.g*. radiology and laboratory), and shortages of nursing or administrative staff [2].

**Fig 1:**
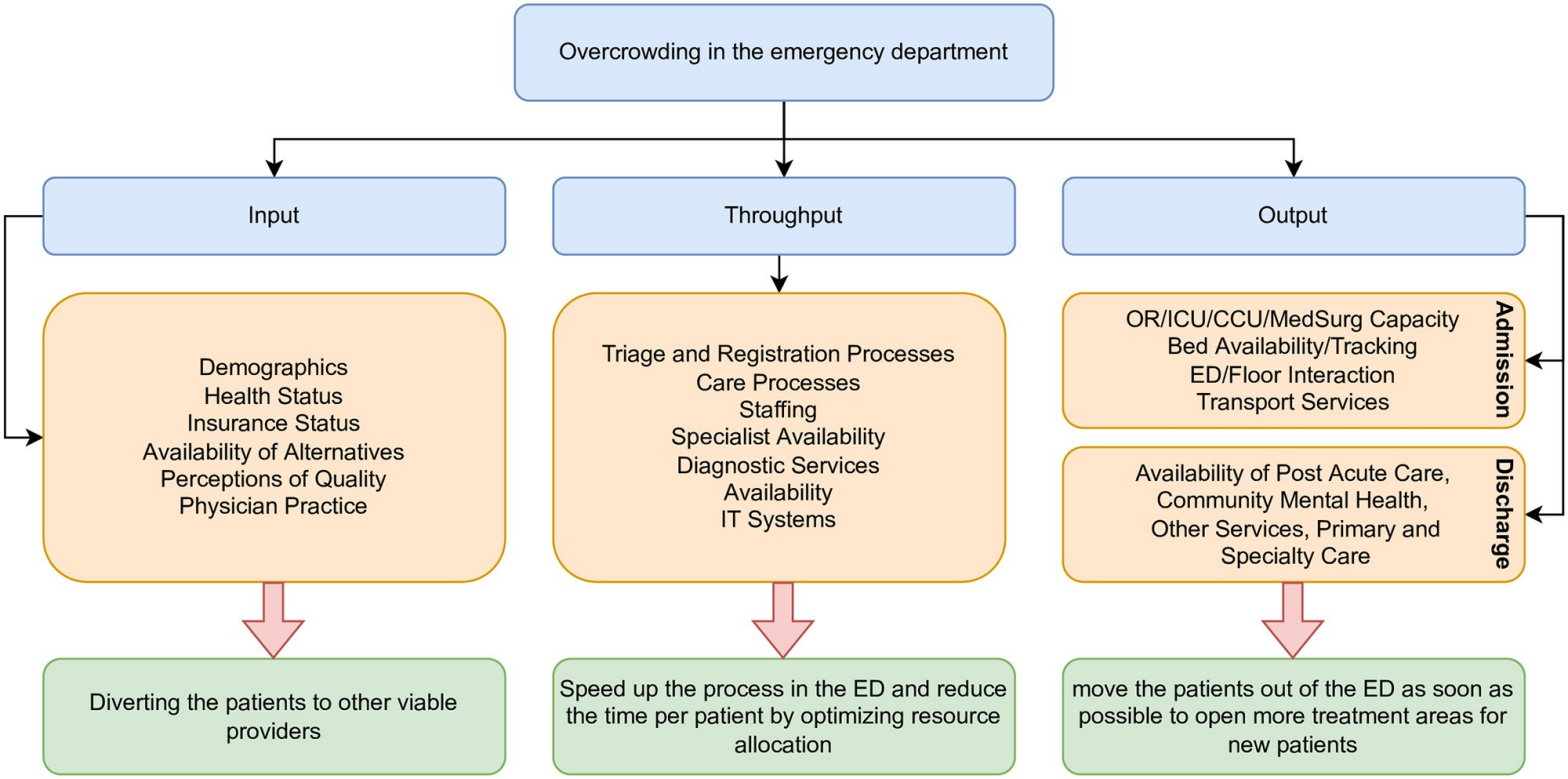
Input/Throughput/Output model of patient flow in the ED, adapted from [5].

While many approaches have been suggested to overcome patient overcrowding in the ED, these methods can be classified into three main strategies named *input*, *throughput*, and *output*. Strategies under the *input* class try to reduce the overcrowding by redirecting patients to other viable providers. *Throughput* strategies attempt to move patients through the ED more quickly by optimizing resource allocation, such as staffing and scheduling changes. Lastly, *output* strategies aim to move patients out of the ED more quickly to provide more treatment areas for new patients [4]. Fig 1 shows the Input/Throughput/Output model [5] of patient flow in the ED and the suggested methods to resolve the overcrowding in each step.

The need for diagnostic imaging may prolong a patient’s length of the stay in ED since this department is often providing services to both admitted patients in the hospital and outpatients in the community. Computed Tomography (CT) scans are a type of radiological procedure that produces cross-sectional images of the body by aiming a narrow beam of x-rays. A common type of CT scan procedure requires the injection of intravenous contrast media to improve soft tissue contrast resolution. These procedures are known as contrast-enhanced CT exams and often require laboratory testing for the patient prior to receiving the contrast material to ensure they have sufficient renal function [6]. Predicting a patient’s need for a CT scan early in the visit may help identify those patients who require laboratory testing before the physician’s initial assessment.

Recently, artificial intelligence has been used in the medical domain; especially, in the ED to predict various clinical and non-clinical procedures [7]. A successful example of using machine learning (ML) in the ED is for the prediction of hospital admissions. Hong *et al*. [8] proposed a model to predict hospital admission using ED triage data with good predictive performance. Hond *et al*. [9] compared tree-based models and logistic regression models in terms of predictive performance to develop a hospital admission model and found that logistic regression performs as well as the tree-based models. The use of ML in the ED is not limited to admissions prediction. Klug *et al* [10] designed a gradient boosting model for predicting early mortality in the ED, Jiang *et al*. [11] developed a model to help with the detection of cardiovascular disease in the ED, and Taylor *et al*. [12] trained and compared ML models to predict urinary tract infections in the ED. All of these studies have shown outstanding results using ML. Finally, Sanchez *et al*. [13] provided a comprehensive review of ML methods applied to triage in emergency services and concluded that ML methods can be a valuable tool in the ED.

Researchers have also shown great interest in using ML and deep learning methods in other medical domains and healthcare purposes such as medical data privacy [14, 15], drug development [16, 17], cancer detection [18, 19], and radiology [20–23]. The advancement of these predictive models has led to better planning strategies, improved patient satisfaction, and increased productivity for nurses and physicians [2]. However, there has been little research in the predictive modeling of radiological services and ED operations. Klang *et al*. [24] proposed a ML model to predict head CT exams in the ED. However, there are no comprehensive studies to examine the prediction of CT exams using triage administrative data. This work aims to develop ML models to predict patient need for a CT scan using triage administrative data. This research has the potential to reduce ED length of stay by identifying those patients early in their visit requiring a CT scan, completing the required laboratory testing and integrating the patient into the existing CT scanner queue.

More specifically, this paper investigates the following main research questions (RQ).

- RQ1: What is the best ML algorithm to predict CT scans and what is the effect of using text embedding methods to encode the chief complaint?
- RQ2: What are the key attributes that predict the ordering of a CT scan?

The remainder of this article is organized as follows. Detailed descriptions of the data and models are presented in the Materials and methods section. The experimental results are presented in the Results section. The performance of the model, its applicability, and its comparison with similar prediction models in the ED are described in the Discussion section. Finally, the Conclusion section provides an overall summary of the problem and the outcomes achieved.

## Materials and methods

### Setting

This study was conducted in the ED at the Thunder Bay Regional Health Sciences Centre (TBRHSC) which is a tertiary care referral centre that serves northwestern Ontario, Canada. The region has a single large urban centre (i.e., Thunder Bay) with numerous rural communities. The region has a population of 232,000 over an area of 526,000km^2^.

The current patient pathway in the ED begins when the patient arrives, undergoes the triage process, and then waits for the physician’s initial assessment (PIA) to determine whether a CT scan is needed. Once the CT scan is done, the patient waits for results and is then either discharged or admitted for further management. By predicting which patient needs a CT scan and integrating this process into a decision support system within the hospital’s IT system, the resources required to complete the CT scan (i.e., laboratory and imaging) can be allocated early to ensure the test is completed quickly. Especially since the patients may wait long periods between the order and acquisition time for the actual CT scan.

**Fig 2:**
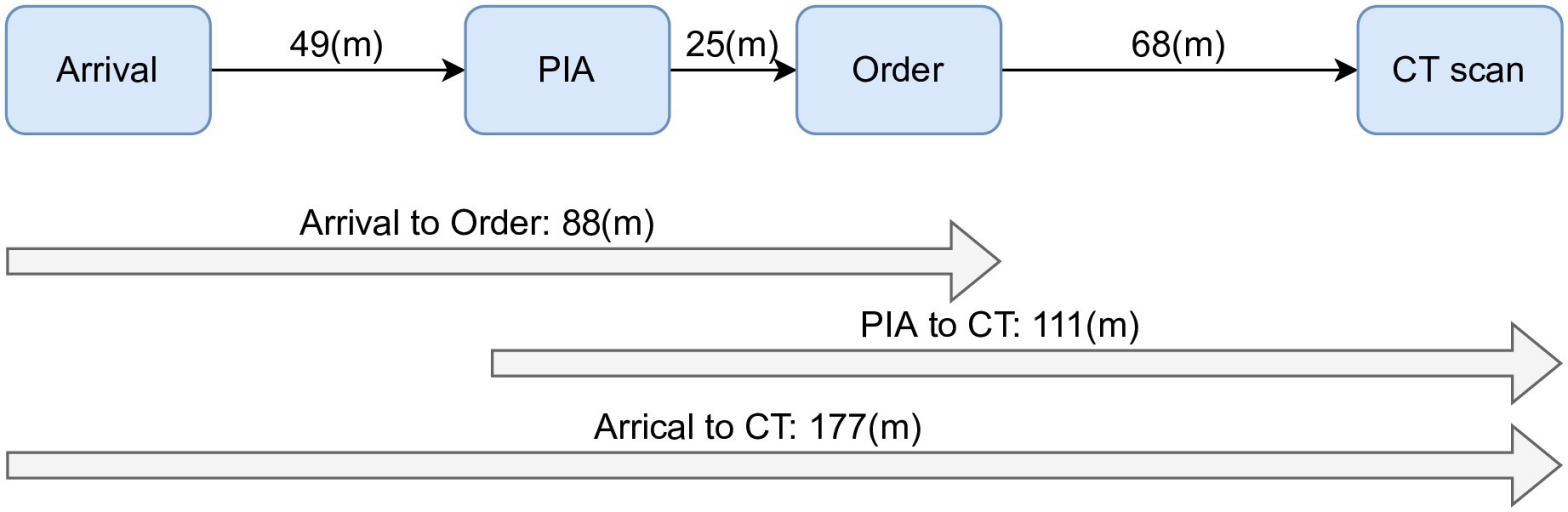
Patient flow in the ED with median waiting time in minutes for each step.

The administrative data revealed that the median waiting time for each patient from arrival until the PIA was approximately 49 minutes. The patients who required a CT scan waited for 88 minutes from arrival until the CT scan was ordered. They had to wait for an additional 68 minutes after the order to begin the actual CT procedure. Fig 2 illustrates the median times for patients to move through each major process in the ED from arrival until their CT scan was completed.

### Data collection and processing

This is a retrospective cohort study of ED triage data routinely collected during a patient’s visit. Ethics approval was obtained from the TBRHSC (REB# 2012102) and consent was not obtained from the patients since the data were analyzed anonymously. For the period of May 2016 to May 2017, 108, 708 patient visits were recorded. The data for this study was anonymized administrative triage information that was collected by the triage nurse. The attributes for this dataset were: age, sex, mode of arrival (i.e., walk-in, ambulance, or other), triage acuity (i.e., from 1 to 5, 1 representing most ill and 5 least ill), chief complaint (i.e., a structured CTAS-compliant textual value fewer than five words entered by the triage nurse), and treatment area (i.e., the area of the ED where the patient is assessed, monitored, and managed after the triage process). The dataset also contained the date and time of patient arrival, service, departure, the PIA, and the admission and transfer time if the patient was admitted to the hospital. CT image data from the diagnostic imaging department was combined with the patient triage data to determine whether a CT exam was ordered for each patient. Each CT image record also contained the date and time of the ordering and the actual time of the procedure. The target variable (i.e., that is to be predicted) for the model was encoded as binary a variable showing whether a CT was performed or not.

**Fig 3:**
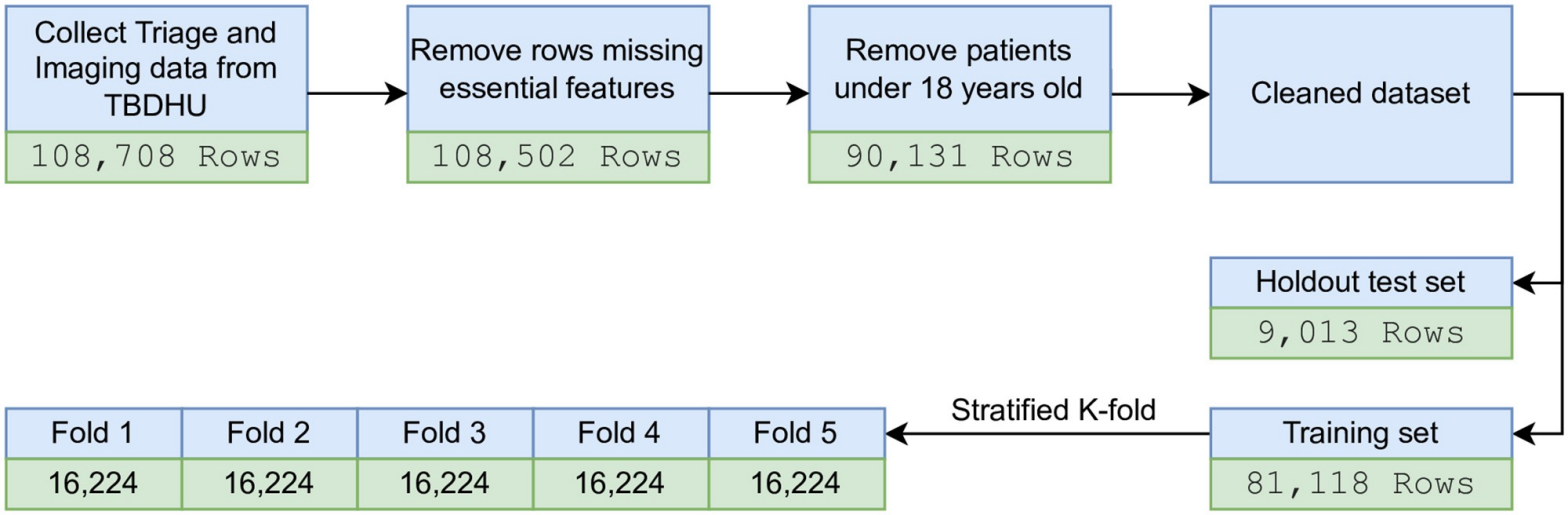
Data preparation steps applied on the dataset.

Patients under 18 years of age were excluded since physicians often apply different criteria to determine which pediatric patient requires a CT scan due to the radiation dose [25] and other factors. Afterwards, the data was cleaned and structured by eliminating rows missing essential attributes such as chief complaint, triage acuity, arrival time, service time, and departure time. For the remaining non-essential attributes with missing values, a new category named *unknown* was introduced instead of dropping the rows. There were also some inconsistencies in the recorded date and times that the authors suspect were input errors. For example, in some cases, the physician’s initial assessment occurred before the patient’s arrival. In such cases, if the time difference was small (i.e., less than 5 minutes), the data point was included in the waiting time calculations; otherwise, it was removed from the waiting time calculations but not the classification task. This step was performed in consultation with the other co-author, an emergency physician. After the data cleaning process, 89, 071 samples met the inclusion criteria. Fig 3 depicts the preparation steps taken and the corresponding number of patients meeting inclusion for each step.

### RQ1: Machine learning models and text embedding

This study examined six common ML algorithms to find the most suitable model for this problem. These algorithms included Logistic Regression (LR), Support Vector Machines (SVM), K-nearest Neighbors (KNN), Multilayer Perceptron (MLP), XGBoost (XGB), and Gaussian Naive Bayes (GNB). Text embedding methods were required to convert the textual chief complaints into a representative numerical vector that can hold the semantics of the original text. Simple statistical methods and state-of-the-art deep learning-based text embedding methods were utilized to generate features from the chief complaint attribute. The embedding methods used in this work were: Bag of Words (BOW), Term Frequency-Inverse Document Frequency (TF-IDF), Word2Vec (V2W) [26], GloVe [27], FastText (FT) [28], and Sentence BERT (SBERT) [29]. In addition to the six embedding methods, the authors have also tried one-hot encoding of the chief complaint as a categorical variable with 165 unique categories.

An exhaustive search was performed to find the best combination of embedding, ML model, and resampling methods, among all of the 210 possible combinations (i.e., 7 embeddings × 6 models × 5 resamplers). Since the dataset was large, it took a considerable amount of time to complete all of the experiments, therefore a random sample with 10% of the training set was chosen for these experiments. Afterwards, the data was compiled into five stratified folds, which preserved the percentage of samples for each class. For each combination, the accuracy and the Receiver Operator Characteristic Area Under the Curve (ROC_AUC) were calculated to compare the performance of these models. The classifiers were then sorted by their ROC_AUC score to find the best model, which were used in further experiments.

### RQ2: Feature importance

Two experiments were designed to study and gain more insights into the importance of the features. The first experiment was a generic approach where the model was trained using only one of the features at a time. Each model was cross-validated and compared to the others to find the most important features. A feature was considered more important if it yielded a higher classification accuracy for the corresponding trained model. The second experiment was based on the feature coefficients of the model. During the training phase, the model associates a coefficient with each of the input features. A higher absolute value for a feature shows that the model is relying profoundly on that feature. The model’s coefficients were computed and used as an indicator of the features’ importance.

### Auxiliary analysis and computational resources

#### Class imbalance

A significant issue in the administrative dataset was the class imbalance observed in the target variables. Among all ED patients, only 13.4% had a CT scan and the number of patients who underwent a CT with contrast was even smaller at 5.9%. Generally, there are two ways to handle imbalanced datasets, either through oversampling or undersampling methods. In oversampling methods, synthetic data from the minority class is generated to balance the number of samples in each class. Whereas in undersampling methods, a random sample of data points from the majority class are removed to balance the classes. This work experimented with two undersampling methods (i.e., random undersampling and nearmiss [30]) and three oversampling methods (i.e., random oversampling, SMOTE [31], and ADASYN [32]) to find a suitable approach.

#### Analyzing the effect of dataset size

A critical aspect in ML modeling is the relationship between the dataset size and performance of the model, which shows whether a model reached its best performance considering the inherent noise in the features [33]. The dataset used in this work incorporated about 90, 000 rows and created the opportunity to experiment with a different number of data points to find the peak performance by gradual increments in the dataset size. To study this relationship, randomly chosen subsets of the data with a different number of rows varying from 1% to 100% of the training data were generated and tested with the top model found in the previous step.

#### Experimental setup

All of the experiments were completed with Python 3.8 using Compute Canada clusters. *Sci-kit learn* [34] and *imbalanced-learn* [35] packages were used for the classification tasks and *NLTK* [36], *gensim* [37], and *sentence_transformers* [29] were used for the text pre-processing and embedding. The nodes used to run the jobs came with 32 Intel E5–2683 CPU cores and 64 gigabytes of main memory. The experiments were conducted in parallel to reduce the computation time.

## Results

### Preliminary data analysis

**Fig 4:**
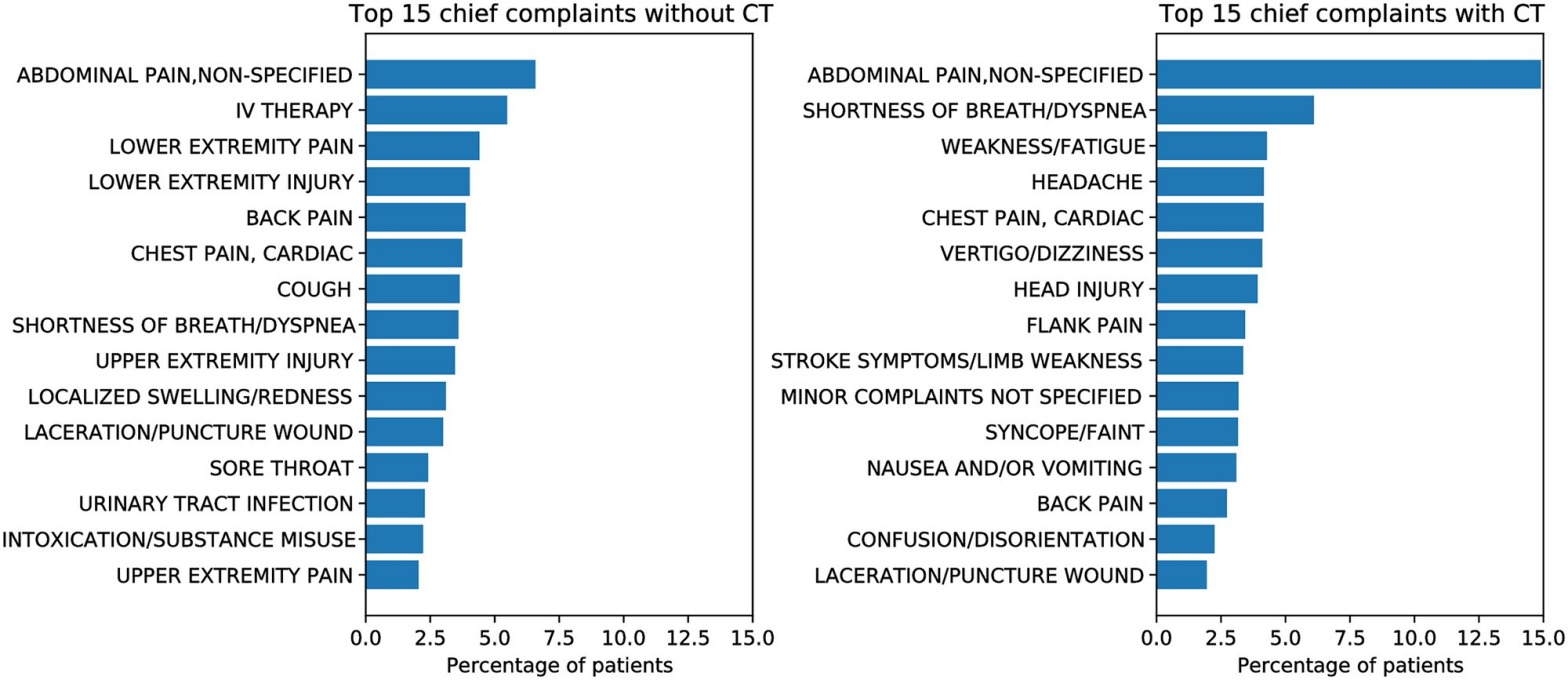
A summary of the top 15 chief complaints for patients who received CT scan (right), and did not receive a CT scan (left).

**Fig 5:**
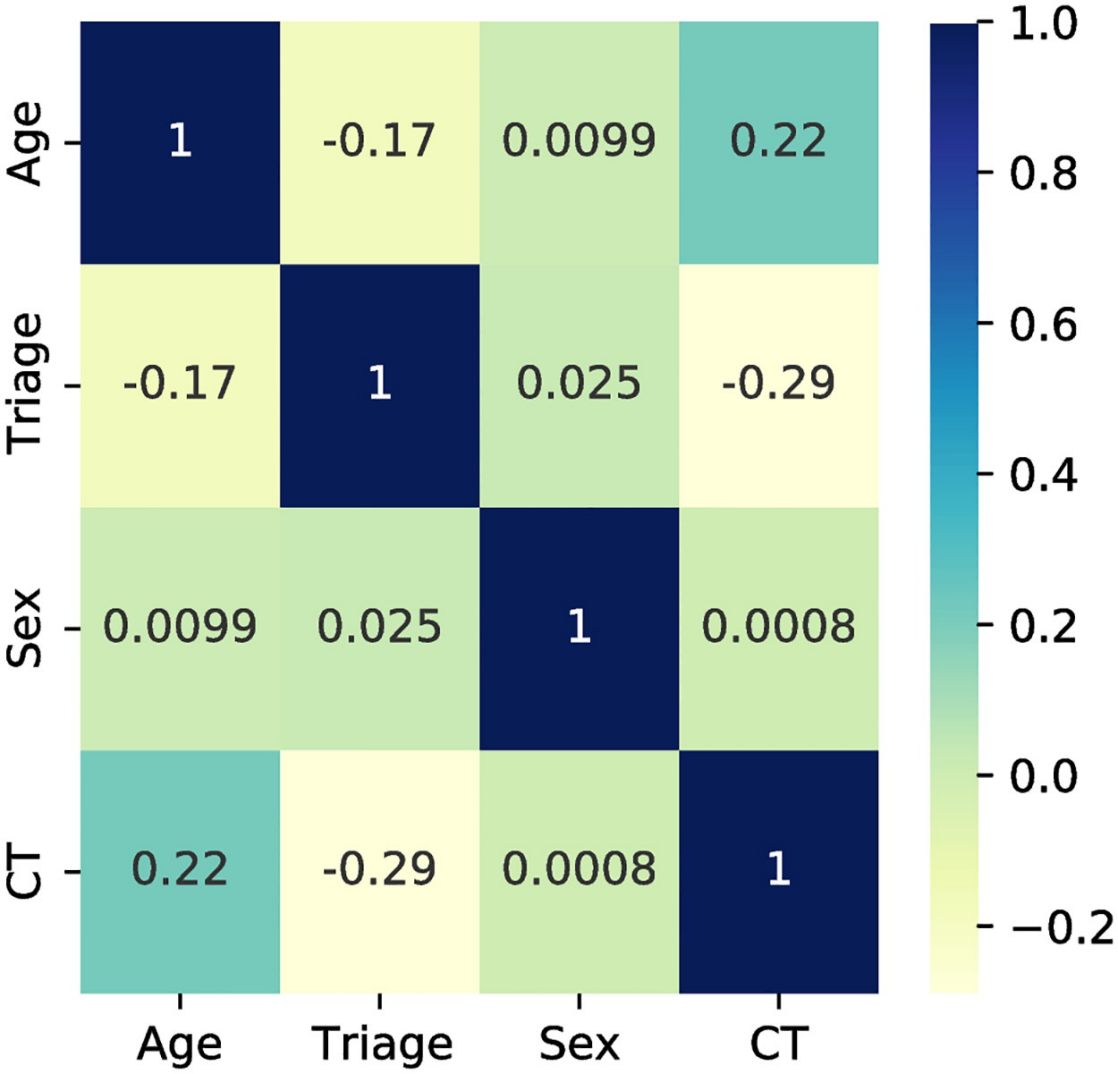
Correlation of continuous features in the dataset.

Exploratory data analysis on the cleaned dataset showed that a CT exam was ordered for 13.4% of the patients. The top chief complaints of patients who received a CT scan are shown in Fig 4. With the exception of *abdominal pain* (which is the most common chief complaint in the ED) and *shortness of breath*, there were no other chief complaints that were common to both populations of patients, indicating that chief complaint may be a good predictor of the target variable. The correlation matrix of the continuous features in the dataset is shown in Fig 5 which demonstrates that older patients were more likely to have a CT scan (i.e., correlation value of +0.22) along with patients having a lower triage acuity level (i.e., correlation value of -0.29) whereas the sex of the patient had almost no predictive value (i.e., correlation value of 0.0008).

### RQ1: Searching for the best pipeline

All possible combinations of embedding, ML models, and resampling methods were tested through an exhaustive search. Table 1 shows the configuration and performance of the 10 best models. The complete table of all results is provided in S1 Table. All of the following experiments used the best model found here, combining sbert embedding, logistic regression classifier, and random oversampling. To evaluate whether the predictive models were statistically different, a t-test was used. However, since the number of models developed in this work was large, not all pairs of models were significantly different. For example, the best and the worst models were considered to be statistically significant (*p*- *value* < 0.0001), while the first two models were not statistically different (*p*- *value* = 0.68).

**Table 1:** Top 10 combinations of embeddings, classifiers, and resamplers to predict CT exams sorted in descending order of ROC_AUC.

Embedding	Classifier	Resampler	ROC_AUC	Accuracy
**sbert**	**logisticregression**	**randomoversampler**	**0.866**	**0.756**
tfidf	logisticregression	smote	0.864	0.767
tfidf	logisticregression	randomoversampler	0.864	0.76
categorical	logisticregression	randomoversampler	0.864	0.757
sbert	logisticregression	smote	0.863	0.762
categorical	logisticregression	smote	0.863	0.763
sbert	mlpclassifier	randomundersampler	0.863	0.78
w2v	logisticregression	randomoversampler	0.863	0.758
sbert	logisticregression	randomundersampler	0.862	0.745
tfidf	logisticregression	randomundersampler	0.862	0.748

To determine the relationship between the text embedding method used to encode the chief complaint and the model performance, an experiment was performed. The model was first trained with only the chief complaint attribute and for each experiment the embedding method was changed. All of the trained models in this experiment achieved the exact same ROC_AUC score of 0.80 regardless of the embedding method showing that the embedding method had little effect on the model’s performance. This may be due to the structured nature of the chief complaints present in this dataset and the relatively small number of unique values for this feature.

### RQ2: Key contributing features

**Fig 6:**
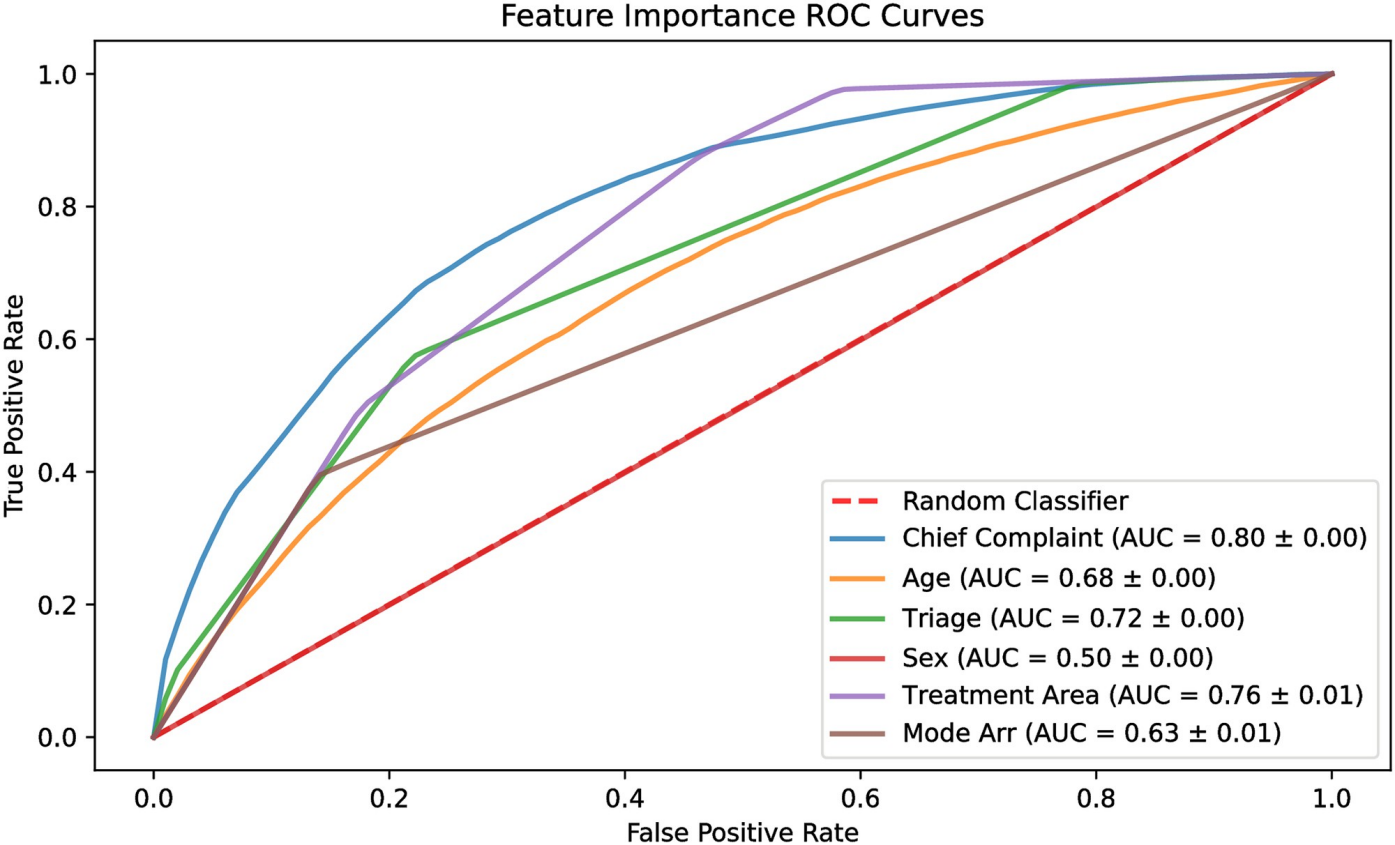
ROC curves of single feature models.

To find the contribution of each feature to the predictive performance of the model, each model was trained using only one feature at a time. Fig 6 shows the ROC curves of these models. Chief complaint had an ROC_AUC score of 0.80, followed by treatment area and triage acuity with 0.76 and 0.72, respectively. The least three impactful features were sex, mode of arrival, and age, having a score of 0.50, 0.63, and 0.68, respectively.

**Fig 7:**
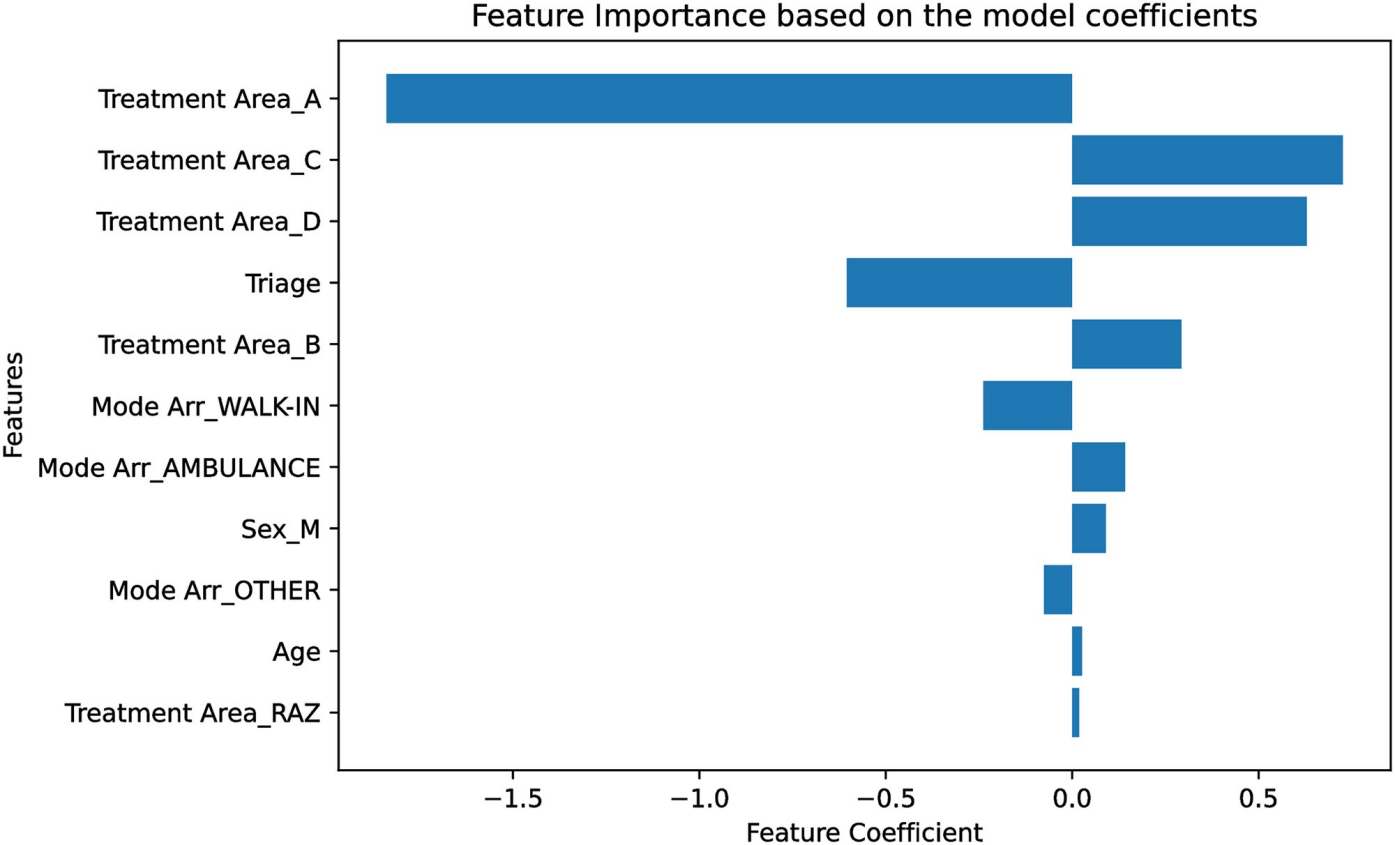
Model coefficients for each feature. A higher absolute value shows that a feature is more influential.

Since the model used in this experiment was logistic regression, the model coefficients were used to determine the feature importance utilizing the complete dataset with all features. A feature with a higher absolute coefficient has more impact on the model output; hence, a more important feature. Fig 7 depicts the model coefficients for all of the features. Since the chief complaint was embedded as a 768-dimensional encoding, it was not included in this experiment.

### The effect of the dataset size

**Fig 8:**
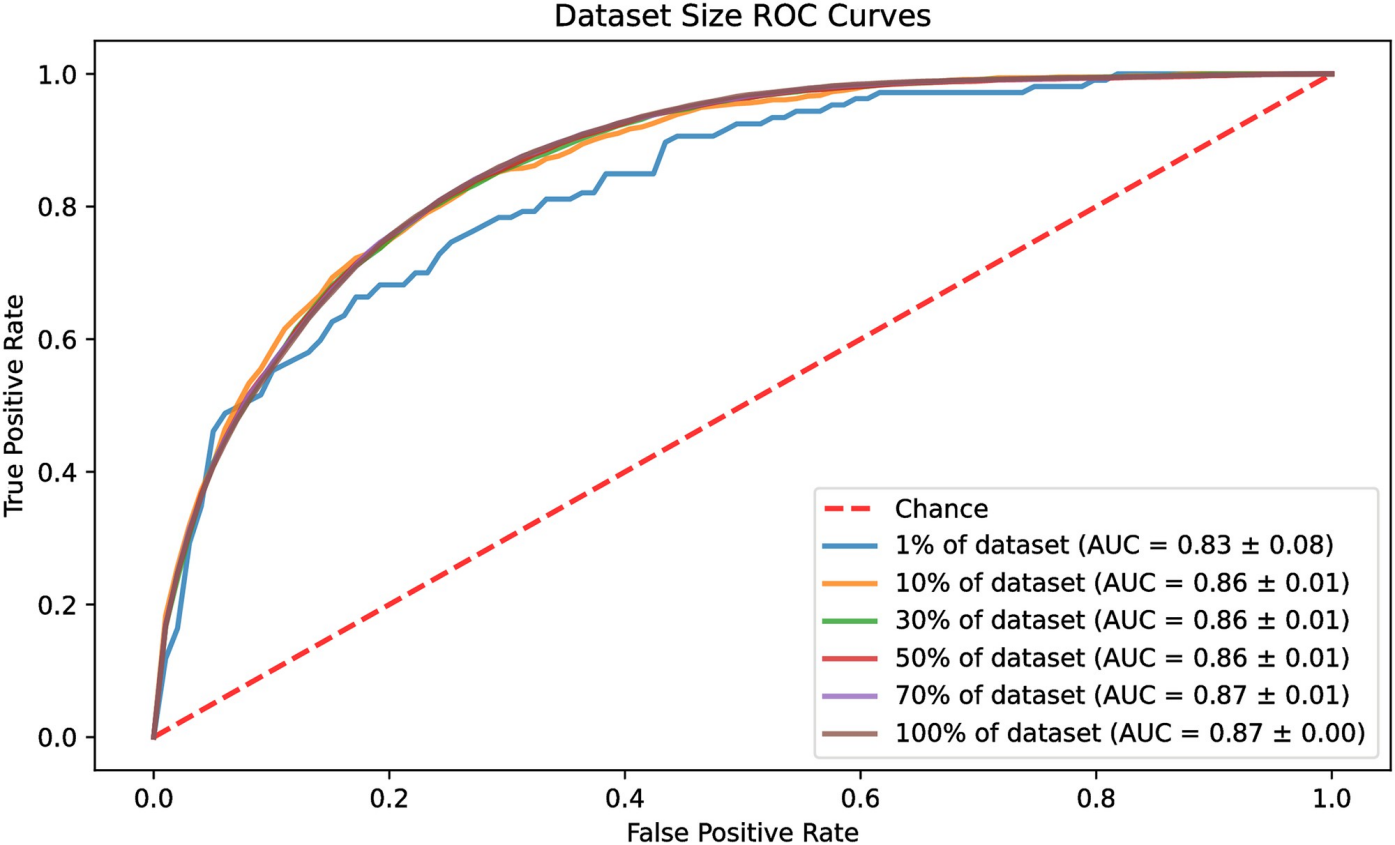
ROC curves of differently sized datasets.

In this experiment, the size of the dataset was increased gradually to find the number of data points required to reach the peak performance of the model. The percentage of the dataset examined was 1%, 10%, 30%, 50%, 70%, and 100%. The results were drawn as ROC curves for each of the data sizes and are shown in Fig 8. The models reached their best performance quickly by using only 10% of the dataset, reaching a ROC_AUC of 0.86. The model with 1% of the dataset performed inferiorly with an ROC_AUC of 0.83, whereas the models with 70% and 100% of the data reached an ROC_AUC of 0.87.

### Hyperparameter tuning

Generally, ML models have a set of parameters that define the architecture of the model. These parameters are referred to as the hyperparameters of the model and can have a substantial impact on its performance. In the case of logistic regression, the most critical hyperparameters are the solver (i.e., the algorithm used in the optimization problem), penalty (i.e., the norm of the penalty function used for regularization), and C (i.e., the inverse of regularization strength; smaller values specify stronger regularization). A grid search was performed to find the best set of hyperparameters for the proposed model. Each experiment was done with 5-fold stratified cross validation and repeated 3 times. Table 2 shows the search space of each hyperparameter along with the top five best-performing models and their corresponding hyperparameters.

**Table 2:** Hyperparameters search space and the top five best-performing models.

Top five models				
Rank	Solver	Penalty	C value	ROC AUC
1	liblinear	l1	100	0.8734±0.0058
2	newton-cg	l2	100	0.8718±0.0052
3	liblinear	l2	100	0.8711±0.0065
4	liblinear	l1	10	0.8704±0.0067
5	newton-cg	l2	10	0.8702±0.0064
**Search space of parameters**				
Solver: newton-cg, lbfgs, liblinear				
Penalty: l1, l2				
C: 100, 10, 1.0, 0.1, 0.01				

### Final model performance

**Fig 9:**
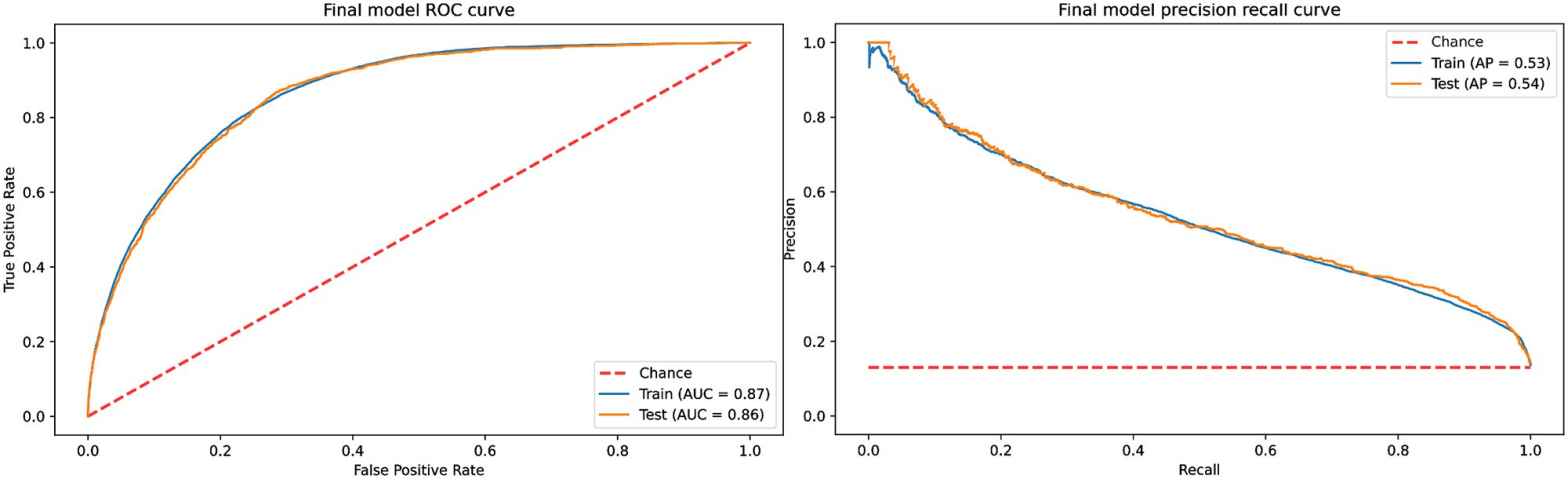
ROC and PR curve of the final model trained using the complete training set.

To verify the final model performance, the whole training dataset was used to train the model, and the performance was verified using the hold-out testing dataset. Fig 9 shows the ROC and precision-recall (PR) curves of the final model for the training and testing datasets. The model yielded a ROC_AUC score of 0.87 and an average precision (AP) of 0.54, respectively. Since the testing data is highly skewed (i.e., 13% positive labels), the proposed model provides a substantial improvement over the baseline AP of 0.13. Using the Youden’s index, the best threshold for the model was calculated as 0.46, which resulted in a specificity of 71% and sensitivity of 87%. The model was also evaluated at different fixed specificity values, and the results are shown in Table 3.

**Table 3:** Performance of the final model on the test set.

	Specificity	Sensitivity	False Positive Rate
Youden’s index	70.9%	87.3%	1:3.5
Fixed specificity 80%	80.0%	74.5%	1:5
Fixed specificity 90%	90.0%	54.5%	1:10
Fixed specificity 95%	95.0%	38.5%	1:20
Fixed specificity 99%	99.0%	16.6%	1:100

## Discussion

This work experimented with different ML techniques and introduced a practical model to predict CT exams in the ED. In this study, multiple ML pipelines were experimented with to determine the most suitable model for predicting whether a patient needed to undergo a CT scan or not. The results showed that a logistic regression model with random oversampling and sbert embedding was the best performing model. The model’s performance on the testing dataset showed that the ROC_AUC score was 0.86. Using Youden’s index, the optimal threshold resulted in a model specificity of 70.9% and sensitivity of 87.3%. With a fixed specificity of 80% and a false positive rate of one patient out of 5, the model’s sensitivity was 74.5%.

EDs and diagnostic imaging departments are experiencing an increased workload that can lead to a negative impact on patients and staff [38]. ML may be a potential solution to improve patient flow in the ED [39]. This study used ML to predict which patient needs to undergo a CT scan or not. Decreasing the overall length of stay in the ED has been shown to have a mortality benefit [40]. A recent study showed that performing CT scans early in the ED visit can reduce the patients’ overall mortality, ICU admission, and median hospital length of stay [41]. The model developed in this study relies on administrative triage data, which is an advantage since it is collected in the initial stage of a patient’s visit to the ED, thus making it a powerful tool for prediction and process improvement. The predictive model developed in this study could be integrated into most ED IT systems. When the triage information for a new patient is collected and entered into the system, the proposed model will be initiated and each patient will be managed according to whether a CT is predicted or not. If a CT scan is predicted, preparations can be made. For example, the imaging department can be alerted and an urgent spot in the queue can be allocated for the patient and laboratory testing for renal function can be completed if necessary. Once the physician assesses the patient and orders a CT scan, they will be ready to have the procedure without further waiting. In the TBRHSC ED, patients wait a median time of 68 minutes from PIA until they receive the CT scan, with this predictive algorithm and process improvements, this wait time can be reduced significantly. Although high sensitivity and specificity is desired from a predictive model, often a tradeoff from one is required to maximize the other. The best ML model in this study has reasonable predictive performance especially in terms of specificity. In this type of system it would be more important to have high specificity to reduce the false positive rate. Since patients predicted to have a CT would be undergoing laboratory testing and would be scheduled in the queue for imaging, it is important not to subject patients to unnecessary laboratory testing and burden the imaging queue with CT scans that don’t occur.

**Fig 10:**
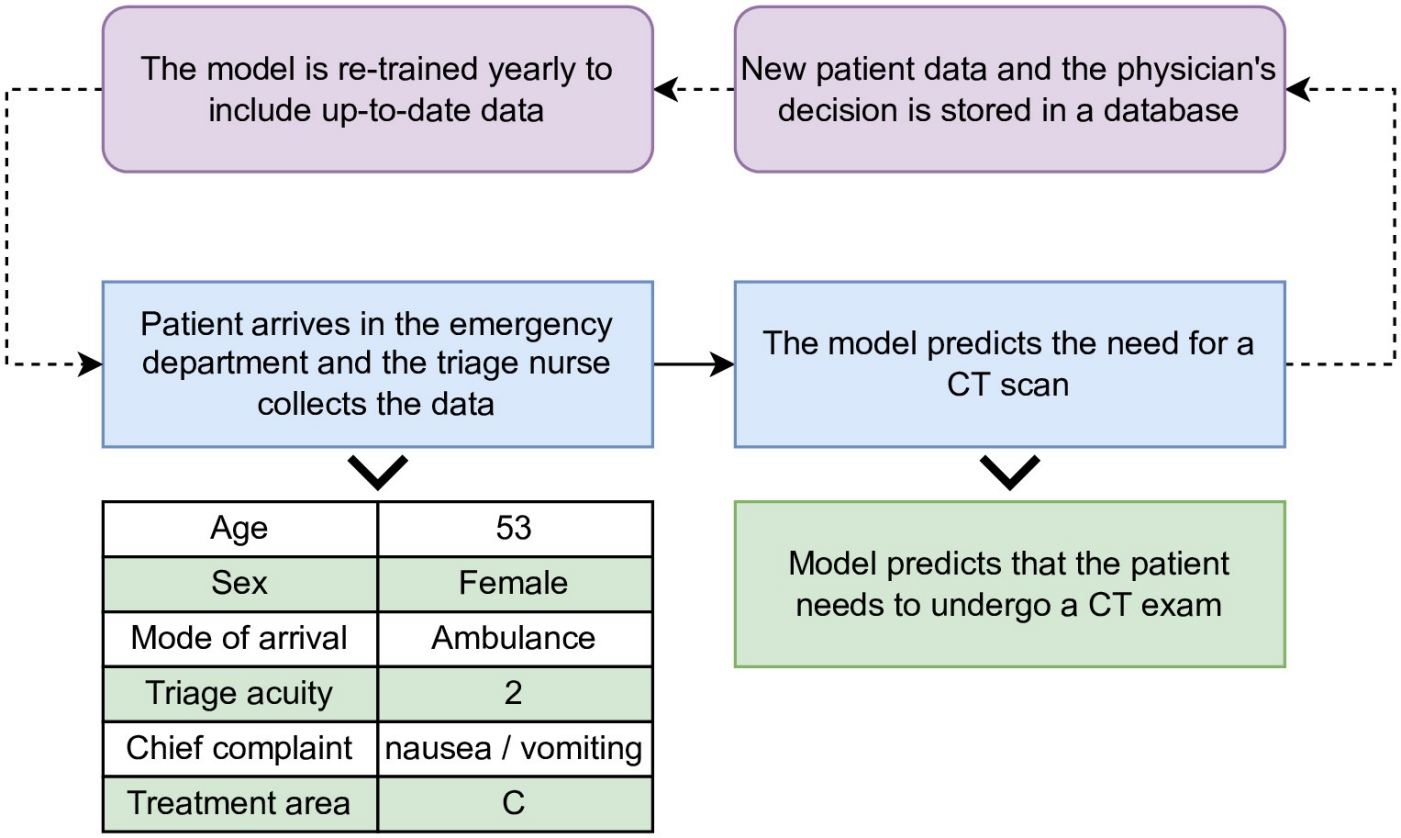
Model integration into the IT system to predict CT exams.

Concept drift is a common issue with predictive models that assume a static relationship between the input and output variables. In the case of CT prediction, the model may become degraded due to variation in physician practice patterns or changes to the standard of care over time. To alleviate this problem, the proposed model can be updated annually using new data collected each year from the ED. This procedure can be integrated into the current model development and evaluation cycle that should occur within the hospital’s IT system. Fig 10 demonstrates how this model can be integrated into the current IT system of the ED and the yearly model training procedure.

To better understand the internal workings of the model and how it predicts whether a patient needs to undergo an urgent CT scan or not, two experiments were designed to rank the features’ importance. The first experiment was based on the single feature performance of the model and the second was based on the model’s coefficients. The top three most important features were found to be the chief complaint, treatment area, and triage acuity, respectively. All three of these features are determined by the partially subjective assessment of the triage nurse based on both quantitative factors (i.e., heart rate, blood pressure, temperature, and other vital signs) and subjective factors (i.e., history of presenting illness, past medical history, and patient appearance). On the other hand, the patient’s sex, age, and mode of arrival were the least important features in determining the CT scan. This result suggests that accurate and consistent triage assessments by the nursing staff would be important for model performance. As well, the factors associated with whether a patient requires a CT or not are not demographic in nature but instead more dependent on clinical factors interpreted by the nurse and thus more difficult to capture in a predictive modelling framework.

Logistic regression has been shown to be an effective modeling algorithm in the ED in previous literature. For instance, Ataman *et al*. [42] used ordinal logistic regression models to predict waiting and treatment time in the ED. Their model achieved an accuracy of 52% and 66% in the waiting and treatment time prediction respectively. They also found that the most important attributes that affect the waiting time in the ED were age, mode of arrival, and ICD-10 encoded diagnoses. Jiang *et al*. [11] compared different ML models such as logistic regression, XGBoost, and random forest to determine the triage acuity of patients with suspected cardiovascular disease. They reported ROC_AUC scores ranging from 0.90 to 0.93 for different learning algorithms. Rahman *et al*. [43] used the decision tree algorithm to predict ED length of stay greater than 4 hours and the accuracy of the model was 85%. Hong *et al*. [8] proposed a model to predict hospital admission. Their model achieved a ROC_AUC score of 0.87 using a deep neural network when the dataset was limited to triage data only. To the best of the authors’ knowledge, there are no other research articles that comprehensively study the prediction of CT exams in the ED. Klang *et al*. [24] introduced a model which could predict head CT exams using the ED triage data, and their model achieved a ROC_AUC score of 0.93. However, their work was limited to non-contrast head CT exams only, and their dataset attributes included patients’ historical data such as previous ED visits and previous CT scans, which may or may not be readily available during the triage process in the ED.

This study has several limitations associated with the data and analysis. First, the data collected for this work was limited to a one-year period for a single hospital and may not be generalizable to other centres. Furthermore, not all hospitals have identical clinical and administrative data available and may have different IT infrastructure to access patient information. The approach outlined in this study could be applied to any ED and may yield better predictive performance if other clinical data is available. Second, the attributes of the collected dataset were limited to administrative triage information. While this set of features can be implemented in a hospital’s electronic health record system, it does not include clinical information which will limit its predictive performance to some degree. It is expected that including more features such as the patient’s history of presenting illness, past medical history, medications, and previous investigations, would improve the model’s performance. However, much of this data is not readily available at the time of triage in many EDs. Lastly, although the proposed model can be of great assistance in predicting a CT scan during the triage process, it still does not account for all of the complexity in physician decision making. Future research in this domain should expand on the algorithmic performance of the model by using deep neural networks for the classification task and include more clinical features such as patient ED visit history, CT scan history, or the patient’s vital signs that likely have better predictive power than basic administrative data.

## Conclusion

Hospitals and EDs are experiencing an increased workload that can negatively impact patients and staff. In EDs, overcrowding is a major problem, and ML might prove useful for improving patient flow. This work introduces a practical approach to predict CT exams in the ED. A total of 210 ML pipelines were experimented with to find the best performing model using triage administrative data. The model developed in this study provides decision support to physicians, nurses, and managers that can be used to align laboratory and imaging resources before the physician’s initial assessment. By aligning resources and planning for the predicted CT scan early in the patient’s visit, ED flow will be improved, patient satisfaction will increase and clinical patient outcomes may be improved.

## Supporting information

S1 Checklist TRIPOD checklist: Prediction model development. (PDF)

S1 Table CT prediction models results. The complete list of the CT prediction models’ results.
(CSV)
